# Holographic Lenses for See-Through Applications Recorded Without Prisms

**DOI:** 10.3390/polym17233164

**Published:** 2025-11-28

**Authors:** Joan Josep Sirvent-Verdú, Tomás Lloret, Juan Carlos Bravo, Cristian Neipp, Andrés Márquez, Sergi Gallego, Augusto Beléndez

**Affiliations:** 1 Departamento de Física, Ingeniería de Sistemas y Teoría de la Señal, Universidad de Alicante, Carretera San Vicente del Raspeig s/n, 03690 San Vicente del Raspeig, Spain; juanc.bravo@ua.es (J.C.B.); cristian@ua.es (C.N.); andres.marquez@ua.es (A.M.); sergi.gallego@ua.es (S.G.); a.belendez@ua.es (A.B.); 2 Instituto Universitario De Física Aplicada a las Ciencias y las Tecnologías, Universidad de Alicante, Carretera San Vicente del Raspeig s/n, 03690 San Vicente del Raspeig, Spain; tomas.lloret@ua.es; 3 Departamento de Óptica, Farmacología y Anatomía, Universidad de Alicante, Carretera San Vicente del Raspeig s/n, 03690 San Vicente del Raspeig, Spain

**Keywords:** photopolymers, holographic lenses, shrinkage, see-through, image quality

## Abstract

Holography offers a wide range of solutions for see-through display applications, where holographic optical elements can act either as mirrors or as waveguide couplers. In the latter case, one of the main challenges lies in achieving efficient mass fabrication. To address this limitation, the use of wavelength shift recording has been proposed, as it eliminates the need for prisms and index matching during the recording process. These elements are typically designed as slanted holographic gratings, recorded using either transmission or reflection geometries. Photopolymers as holographic recording materials are a promising solution for such applications because of their attractive optical properties. However, their inherent volume changes affect the optical performance of the recorded elements. In this paper, we propose the use of holographic lenses as wave couplers, which enables control over additional parameters such as magnification and optical aberrations. We analyze the limitations of this recording approach when prisms are not employed, and we investigate the influence of photopolymer shrinkage on hologram quality, comparing lenses recorded using transmission and reflection holography with different focal lengths.

## 1. Introduction

See-through displays are one of the most appealing fields to invest money in for technological companies such as Apple [1], Samsung [2], Bosch [3], and Microsoft [4]. As a result, in recent years, many patents have been developed to solve different problems associated with this technology [5,6,7,8].

Holography is an important tool in many of these designed visual schemes and displays, and two main holographic optical elements related to these have been used, holographic mirrors [9] and holographic wave couplers [10], based on slanted holographic gratings mainly. Both come with inner bottlenecks: the mirrors are affected by chromatic aberration when dealing with RGB images, and the recording scheme for the couplers relies on a very oblique angle to permit the image to be guided through the substrate using the principle of total internal reflection (TIR). There are two principal solutions to achieve such tilted angles. First, by using prisms and an index matching system, such tilted angles can be introduced, even providing a continuous focus adjustment [11], but it comes with the cost of limiting its mass production. Recently, a wavelength shift between the recording process and the replay has been proposed to obtain the TIR effect during hologram playback. With this technique, our research group has provided pioneering evidence in the design of both transmission [12] and reflection [13] recording schemes. In addition, the adaptation of the recording geometry to different photopolymer recording materials has been studied by slightly changing the slant angle and spatial period of the gratings [14]. An environment-compatible photopolymer has been used as the recording material for these holographic optical elements [15], proving that this design is compatible with multiple sources and versatile. Similar designs have been proposed to implement this type of element in sensing applications [16] by using a multiplexing recording scheme. Other groups also contributed to this solution with interesting studies [17,18,19,20] about the design of slanted holographic gratings in transmission and reflection, including RGB proposals. Lately, polarization volume gratings have also been studied as a strategy to improve the uniformity of the propagated image [21].

The recording material used for this goal is typically a photopolymer-based film, due to its well-known properties such as its low price and stability, adjustable thickness, high transparency, and high values of refractive index modulation [22,23]. There are many families of photopolymers, and a couple of companies have created commercial layers with specific properties (such as optical thickness, absorption wavelength band, etc.).

Some important characteristics of photopolymers are the shrinkage and swelling that can occur during hologram formation, which correspond to a thickness variation in the material film after the exposure step. Specifically, shrinkage is due to the more compact structure that arises from the monomer molecule bonds during the polymerization process, while swelling is due to the migration of molecules described by Fick’s law [24]. The value of shrinkage in photopolymers can vary from 1% to more than 10%, which can be quantified with different methods [25], such as by analyzing Bragg detuning [26] and considering that shrinkage only affects one of the components

(1) $$K=k_{r}-k_{o}$$

of the grating vector K [27,28]. In detail, for a sinusoidal grating, K is given by the wave vectors $k_{r}$ and $k_{o}$, which are, respectively, the reference and the object beam during the recording process, whose modulus is $2\pi n_{0}/\lambda_{0}$, related to the wavelength of the recording light $\lambda_{0}$ in air and the refractive index of the recording medium $n_{0}$.

To find the value of K after recording, it is more accurate to measure different Bragg’s conditions and obtain a completely new K vector [28]. Shrinkage depends on the material’s constituents, the recording process, and the post-bleaching/curing method, and its influence is also application-dependent. It has been reported that photopolymers like Bayfol [29], with a refractive index of $n_{0}$ = 1.505, only have a value around 1.5% in transmission and reflection schemes. PVA/AA presents 3% for spatial frequencies of 2000 lines/mm, and a value of 5% has been mentioned in other applications of Biophotopol, a photopolymer with a sustainable design [25]. Also, other effects like the bending of interference planes can be observed in holographic recording materials [30].

The recording of slanted sinusoidal gratings has some limitations; thus, some patents [5,6,7,8] propose the recording of holographic gratings with prisms and the recording of holographic lenses also with prisms to design optical systems with different properties, like by adapting the magnification of the guided images. Additionally, the quality of the recorded lens is an important factor to analyze in this kind of see-through system [31,32,33]; to achieve this goal, we used a Hartmann–Shack (HS) sensor. The quality of a lens can be measured using different methods and coefficients such as Zernike coefficients, Seidel coefficients, Marechal tolerances, root mean square (RMS), peak to valley, critical fraction of the pupil, etc. [34]. In this work, we chose a Shack–Hartmann wavefront sensor [35], but we also could have chosen a CCD sensor, following the steps presented in [34,36] to obtain the aberration parameters and quantify the quality of the holographic lenses (HLs) recorded in the photopolymer. Employing a HS sensor provides better results in terms of lens resolution than when using a CCD sensor, as analyzed in [37].

In sum, we propose and analyze the recording of HLs, according to a wavelength shift. These elements can control some properties of the final image, such as magnification and access to the Fourier plane, and partially control image aberrations, even trying to tailor them with a pre-compensation method by using a wavefront modulator [38,39]. When the optical system is composed of lenses, convergent or divergent, the control of the focal lengths is crucial to avoid blurred images and to know where the Fourier plane is located to be able to conduct Fourier image processing.

Therefore, this paper is organized as follows: Firstly, experimental methods regarding the recording process and the aberration inspection are stated. Next, the features of the shrinkage model of photopolymers are applied to the study of holographic lenses. Therefore, we provide evidence of the successful experimental recording of the holographic lenses, the calculations related to the variations that the shrinkage introduces in such elements, and the analysis of quality, through modulation transfer function (MTF) measurements and aberrations.

## 2. Materials and Methods

### 2.1. Holographic Recording Setup and Geometry Design

**Figure 1:**
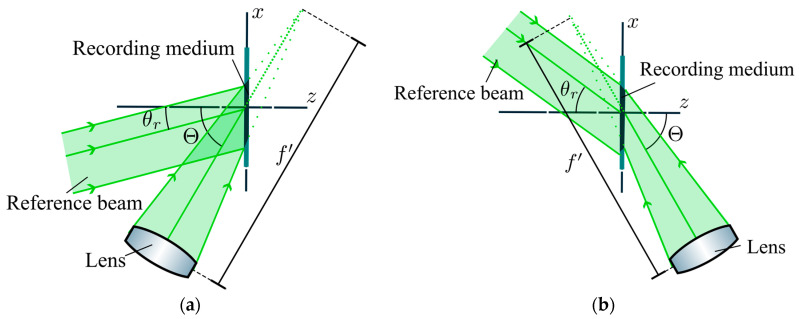
A schematic representation of the incident beams in the recording process in (**a**) transmission $(\theta_{r}$ = 4.8° and Θ = 72.8°) and (**b**) reflection geometry ($\theta_{r}$ = −30.0° and Θ= 63.2°).

We use a holographic recording setup where a continuous-wave laser (Spectra Physics, $\lambda_{0}$ = 532 nm, Santa Clara, CA, USA) is split into two arms through a beam splitter, both spatially filtered and collimated to serve as the recording beams [28]. Then, a refractive lens is placed before the object beam reaches the recording medium, as Figure 1 depicts. In the case of slanted sinusoidal gratings, the exposure scheme dictates which wavelength is to be used, 633 nm for the transmission elements and 473 nm for the reflection counterparts [13], where the recombination of angles for holographic lenses to be used as wave couplers depends on the wavelength shift. In the case of transmission, the beam $k_{r}$ impinges on the recording medium with +4.8° and the beam $k_{o}\;$ with +72.8°. For the reflection case, beam $k_{r}$ impinges at −30.0° and the beam $k_{o}$ with 63.2°. In this case, the half aperture of the object beam is 5 mm.

**Figure 2:**
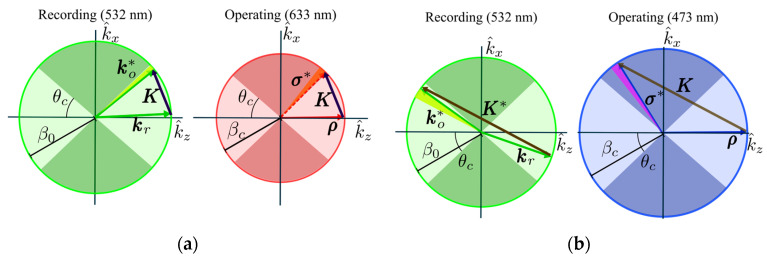
Ewald’s spheres for recording geometries in (**a**) transmission and (**b**) reflection for the HLs. The highlighted arc next to the object/diffracted beam depicts every vector within the span of the holographic lens. Hence the superscript ∗ denotes a representation of such span with one of its vectors. Shadowed circular sectors, taking into account the critical angle, represent the TIR regions in the material.

The corresponding representation in the Ewald sphere of the proposed geometries is presented in Figure 2, using Equation (1). In each case, both the recording and operating spaces are shown, whose axes correspond to the grating vector components in the reciprocal space ${\hat{k}}_{z}$ and ${\hat{k}}_{x}$, respectively, and in both, the whole span of diffracted beams is highlighted in a shadowed arc. This fact rests upon the assumption that in each region, the lens can be treated as a planar grating [19], whose properties change continuously between extremal rays.

ρ and σ denote the wave vectors in the operating space, whose wavelength is different from the recording one. This shift is essential to our purpose because it permits us to use this recording process without prisms and ensures that every ray, given by each $\sigma^{*}$, fulfills a near-normal Bragg condition and a TIR propagation (as its corresponding angle is greater than the critical angle in the system $\theta_{c}$).

In our experiments, we recorded focal lengths of 8 and 12 cm. To record holograms, we used the Bayfol HX200 photopolymer fabricated by Covestro AG (Leverkusen, Germany) [29], with a physical thickness of 16 μm. After recording, a curing process of only 5 min is performed as a post-processing mechanism to terminate any reminiscent photopolymerization in the material so as to fix and maintain its properties. Then, the first characterization is conducted by means of the angular response, as measured from the relative efficiency as a function of the playback angle, given by the rotating mount where the sample is placed.

**Figure 3:**
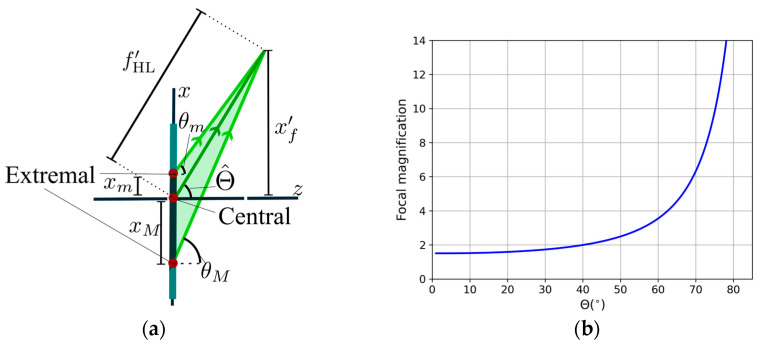
(**a**) A schematic representation of the propagation angles and extremal rays when considering a holographic lens in air. (**b**) Focal magnification as a function of the object angle Θ from the comparison between the substrate and air propagation.

Compared with previous studies, as in [15], the angle modification in the recording geometries is due to the lens and depends on its focal length and beam size, as shown in Figure 1 and Figure 3. Also, it is ensured that for each operating wavelength, the whole span of diffracted rays fulfills the TIR condition. Therefore, considering the two extremal rays, as in Figure 3a, denoted by subscripts m and M, we can obtain the focal length along the substrate considering the shift using

(2) $$x_{f}^{\prime }=\frac{x_{M}\cdot \tan\theta_{m}-x_{m}\cdot \tan\theta_{M}}{\tan\theta_{m}-\tan\theta_{M}},{f_{HL}}^{\prime }=\frac{x_{f}^{\prime }}{\sin\hat{\Theta }}\;$$

where *x* is its position in the hologram axis, so the size of the HL is $D=x_{M}-x_{m}$, and θ is the corresponding propagation angle. Then, the total focal length ${f_{HL}}^{\prime }$ is measured from the center of the HL, following the convention in Figure 3a.

In this sense, it is important to note that the differences in the focal length in air and inside the substrate depend strongly on the recording angle of the beam. There is an increase in the focal length in the substrate up to 6 times for the transmission geometry, as Figure 3b shows, where the magnification of the focal length inside the substrate with refractive index $n_{0}$ = 1.505 is represented. The high tilted angles produce a high degree of magnification in focal length. To be sure that all the rays fulfill the condition of TIR and the diameter of our image is 20 mm, the shorter focal length, in air, that we can use at 633 nm is around 40 mm, and for this value in the substrate, the focal length corresponds to 204 mm approximately. These are essential facts if these couplers are to be used in a waveguide combiner, where an appropriate focal length must be recorded as a function of the distance between the in- and out-coupler.

With respect to the lens quality measurements, we follow the steps described in [34]. The wavefront sensor used in this work was a Hartmann–Shack WFS30-5C model from Thorlabs (Bergkirchen, Germany). This instrument is composed of a 1936 × 1216 pixel CMOS camera with an active area of 11.34 × 7.13 mm^2^, an array of microlenses with a pitch of 150 μm, and an effective focal length of 4.1 mm. The recorded holographic lenses have a height of 20 mm, which is not symmetrical based on the central and extremal points that can be seen in Figure 3a. Nevertheless, we only worked with a beam with a diameter of 6 mm, due to the limitation imposed by the complementary metal–oxide–semiconductor (CMOS) of the HS wavefront sensor, so our results are subjected to this constraint.

### 2.2. Shrinkage Approximate Model for Lenses

**Figure 4:**
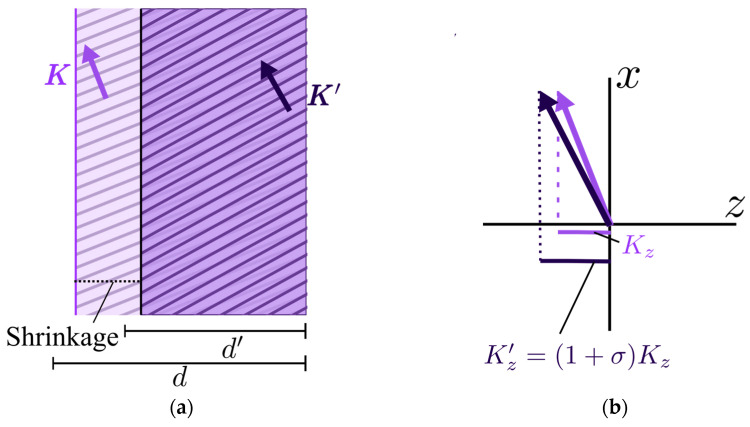
The fringe rotation shrinkage model: (**a**) **a** change in the fringe’s structure due to the reduced thickness σ of the material; (**b**) a comparison of the grating vectors (resized to a larger scale): the *x*-vector remains constant, whereas the *z*-component changes according to the shrinkage value.

Our presented model is based on some of the shrinkage models used for sinusoidal gratings by different researchers [19,25,26]. In general, when the shrinkage of the photopolymer takes place, given by the relative thickness difference σ, it is considered that the vertical component of the grating vector $K_{x}$ remains constant and its horizontal counterpart $K_{z}\;$ increases linearly with σ, as given in Equation (3). This is known as the fringe rotation model [25], which lies upon the assumption that fringes are not altered in the back side of the surface, as shown in Figure 4a.

(3) $$K_{z}^{\prime }=\left(1+\sigma \right)\cdot K_{z}$$

The shrinkage effect on the modification of the original grating vector K to the modified one $K^{\prime }$ in this model is graphically depicted in Figure 4b. This means that the reduced thickness implies, through the increased modulus K, a smaller spatial frequency.

Some groups have demonstrated that this assumption is not completely true, and sometimes changes in the *x*-component [25] can also be observed, probably due to bending effects in the grating [30]. In this sense, it is more precise to measure the two main Bragg angles and, if it is possible, the second-order Bragg angles to determine the state of the K-vector after recording and the variations suffered during recording and the curing process. In the case of the present work, it is not possible to access the second Bragg angles, due to the high slanted geometries and the fact that one of the beams is guided by the TIR effect; therefore, we use the model of the conservation of the *x*-component of the recorded grating vectors.

As we present in Figure 2, the Ewald representation of a holographic lens can be understood as the spatial span of grating vectors around the central angle, the width of this interval depending on the focal length of the refractive lens used and the diameter of the beam in the recording step. Taking this local approximation into account, the shrinkage model is well-suited to be applied in these holographic lenses recorded in photopolymers. This is the method we use to compute the focal length changes within this model: given the shrinkage σ, we compute the modified grating vectors along the spatial span of the lens with Equation (3); hence, the propagation angle of the extremal rays can be recovered, taking into account that the reconstruction beam may be of a different wavelength than the recording ones. Thus, with these angular span values, we compute the focal length along the substrate with Equation (2).

By using this model, we can simulate how shrinkage and wavelength shift in transmission and reflection geometries, this will be an important tool for designing optical systems [5], where we need high precision to avoid blurred systems. It is important to note that the method based on Ewald spheres is just an approximate method considering that Bragg’s condition is only fulfilled for the central value of K. The extremal values of the interval do not fulfill Bragg’s condition; thus the use of Kogelnik wave theory [40] is not the most accurate method for analyzing diffracted light, and other theories like beta-value [41,42,43] are more accurate in predicting off-Bragg behavior [44,45]. In our case, nonetheless, the deviation in these angles is small, so we can stick with the simplest Kogelnik coupled wave theory (CW) that is accurate enough.

**Figure 5:**
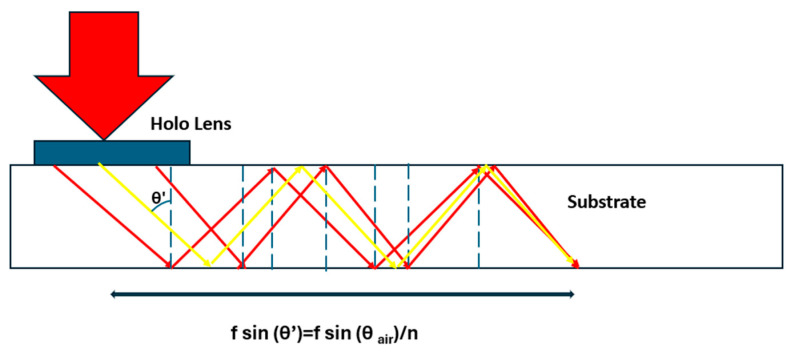
The beam focalization scheme inside the substrate, where multiple TIRs are included, from central (yellow) and extremal (red) points.

The focalization inside the substrate is presented in Figure 5. The collimated beam with image information impinges in the holographic lens and is diffracted by fulfilling the state of TIR inside the substrate. If we want to operate, for example, a 4-*f* system between the in-coupler lens with $f_{in}$ and the out-coupler lens with $f_{out},$ then the distance between the two holograms must be set accordingly as the correct sum of the focal lengths as measured in the substrate.

## 3. Results

In this section, we present, firstly, the simulations realized with our shrinkage model for holographic lenses acting like couplers for see-through systems both in transmission and in reflection geometries. To check our model, we record holographic lenses with a wavelength of 532 nm both in transmission and reflection geometries. If the readout beam is small, we can consider that K is locally constant, and we can simulate the behavior using coupled wave analysis and obtain parameters like refractive index modulation and optical thickness. Lastly, we use a Hartmann–Shacks sensor to measure the aberration of the wavefront [34] after the manufacturing process of these wavelength-shifted holographic lenses.

### 3.1. Shrinkage Model Simulations

Using the shrinkage model for lenses, we computed the change in the focal length associated with different values of shrinkage using Equations (2) and (3). In detail, a new set of grating vectors was computed with Equation (3), which correspond to the shrinkage effects within the whole span of the HL. Then, after the appropriate wavelength shifting, we obtained the new collection of $\theta_{m}$ and $\theta_{M}$ through the corresponding Bragg condition so that Equation (2) yields the new focal length of the system. In Table 1 and Table 2, respectively, we use the recombination of angles given in the experimental section for the transmission and reflection cases.

In our simulations using the fringe rotation model, it is important to note that shrinkage helps us to achieve the TIR effect in transmission, as the diffracted angle inside the material for the operating wavelength increases. On the other hand, for the reflection case, there is a certain value of shrinkage above which the TIR effect will disappear, as follows from the decreasing trend in Table 2, but this range is far above the typical shrinkage values that occur during experiments. Thus, in our case, the TIR effect is maintained in every feasible scenario.

Moreover, we find this analysis essential for characterizing these couplers. In the first place, the shrinkage effects in the HL will help to explain deviations and some effects in the angular responses, meaning that for a fixed operating wavelength, there will be a change in the maximum achievable efficiency. Secondly, it will limit the system design if these couplers are to be included in a waveguide combiner [29], as the distance between the in-coupler and out-coupler will be affected. In this case, a previously established compensation method is needed [46].

**Figure 6:**
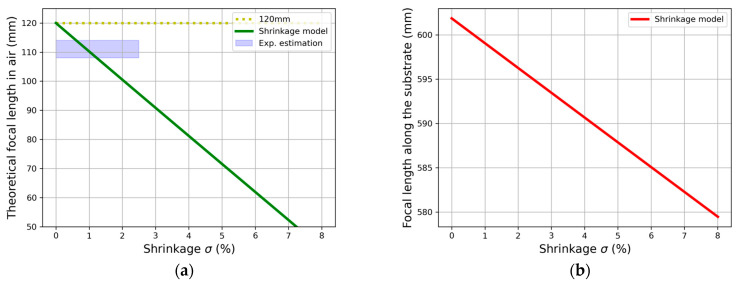
Qualitative estimation of shrinkage model in focal length of transmission HL of 12 cm: (**a**) using $\lambda_{c}=\lambda_{0}=532$ nm and its comparison with experimental values and (**b**) corresponding focal length along substrate when operating wavelength is used (633 nm).

To estimate the shrinkage effects in the holographic couplers, we can directly measure the focal length of the HL by using the recording wavelength. By doing so, we avoid TIR propagation within the substrate, so we can directly measure the focal length as the distance between the center of the HL and the focal point (in air). Figure 6 centers around the case of a transmission HL.

This serves as an experimental validation of the model, as Figure 6a shows when a focal length of 120 mm has been recorded: the reduced value of ~110 mm is coherent with the usual shrinkage in this material (1.5%). Figure 6b presents the numerical results of Table 1 when the focal length is changed, so we can conclude that the recording focal length and the HL dimension are more crucial factors for the design of these elements than shrinkage itself.

### 3.2. Angular Response of Holographic Lenses

In this section, we analyze the recorded lenses using Bayfol as the holographic recording medium and the setup in Figure 1. Herein, we present the angular responses of the transmission efficiencies of the HL as a function of the playback angle $\theta_{p}$, as they can be placed in the original recording setup with a rotating mount. By using a narrow beam, we avoid huge variations in the spatial frequency, so the HL can be approximated as its central grating.

**Figure 7:**
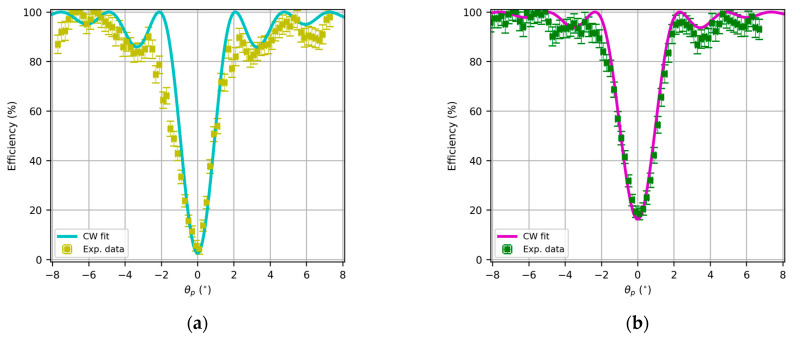
Angular response of transmission HL with focal length in air of (**a**) 8 cm and (**b**) 12 cm, replay with 532 nm, and fitting using Kogelnik coupled wave theory.

In this sense, it is important to remark that the maximum shrinkage calculated by Covestro for this photopolymer is 1.5%. In this sense, if the angular or spectral response is fitted using coupled wave theory, the first approximation of the refractive index and optical thickness can be performed, which, respectively, are d = 16.0 μm and $n_{1}=1.62\cdot 10^{-2}$ for Figure 7a and d = 15.9 μm and $n_{1}=1.84\cdot 10^{-2}$ for Figure 7b.

The reflection lenses are characterized by a high spatial frequency, in our case, around 5600 lines/mm. In this case, the holographic optical element is placed on the back surface of the substrate to guide the substrate along the diffracted order that initially travels from the hologram to the substrate. In this case, it is not possible to produce overmodulation effects [42,47] that decrease diffractive energy, so diffraction efficiency asymptotically increases to 100% when refractive index modulation increases.

**Figure 8:**
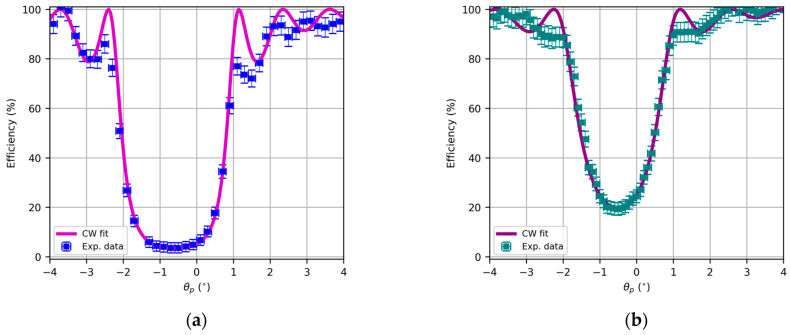
Angular response of reflection HL with focal length in air of (**a**) 8 cm and (**b**) 12 cm, replay with 473 nm, and fitting using Kogelnik coupled wave theory.

Again, the CW fittings yield an estimation of the grating formation parameters, in this case d = 13.2 μm and $n_{1}=2.1\cdot 10^{-2}$ for Figure 8a and d = 11.7 μm and $n_{1}=1.4\cdot 10^{-2}$ for Figure 8b.

### 3.3. PSF Analysis

**Figure 9:**
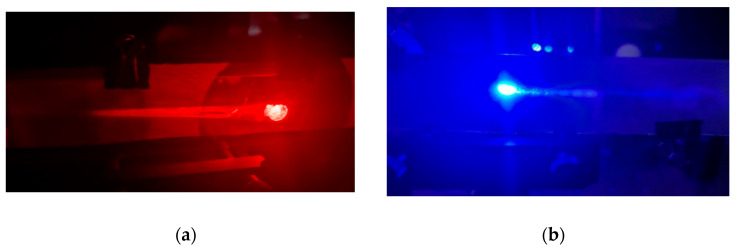
Photographs of the holographic lenses with a recording focal length of 80 mm in air, guiding the corresponding operating wavelength (**a**) transmission HL with $\lambda_{c}=633$ nm and (**b**) reflection HL with $\lambda_{c}=473$ nm.

As Figure 9 shows, the converging beam is trapped inside the substrate for both recording schemes. Therefore, we must measure the quality of the HL with the recording beam once the material is cured, as then the diffracted beam can be directly measured with the HS sensor.

**Figure 10:**
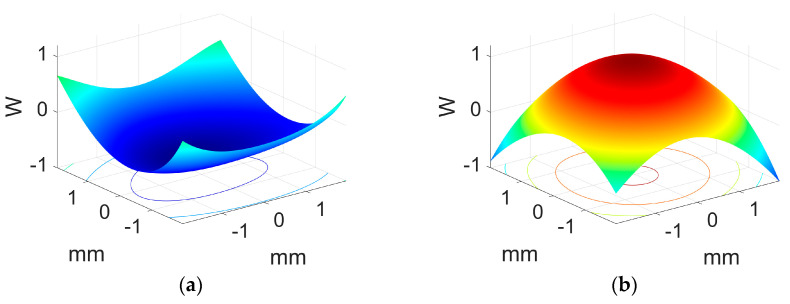
The wavefront aberration function as measured by the HS sensor for the HL with the (**a**) transmission and (**b**) reflection recording geometries in Figure 1.

Using this TIR-based design of the HLs, we measured aberrations and the wavefront form using a recording light, where the light was not guided along the substrate. The measurements were performed using an HS wavefront sensor. The HS sensor is based on a Hartmann screen with uniformly distributed holes, which produces an impact diagram on the image plane. To overcome low-illumination conditions, the HS sensor introduced a lens in front of each hole, thereby improving the measurement accuracy. In this study, the HS wavefront sensor was used for the first time to quantify aberrations in holographic lenses. The HS measures the Seidel coefficients, and thus we obtained the MTF of the lens and after the wavefront aberration following the steps described in [34]. To estimate lens resolution, the HS sensor presents closer agreement with the theoretical predictions than other methods based on CCD sensors [37]. The experimental setup we used is like the presented one in Figure 4 in reference [34] to yield the wave aberration function in the image plane, which we show in Figure 10 for the transmission and reflection HLs, respectively, represented based on the coefficients measured with the HS sensor. Considering insights regarding the quality of the holographic lenses in this representation, we can underline that the values of the aberrations are similar to those obtained in other works for positive lenses, like those recorded here. It is important to note that in the previous works mentioned, ones with negative results presented fewer values for aberrations. Noticeably, the waveform aberration function has the same magnitude in both cases but different forms.

**Figure 11:**
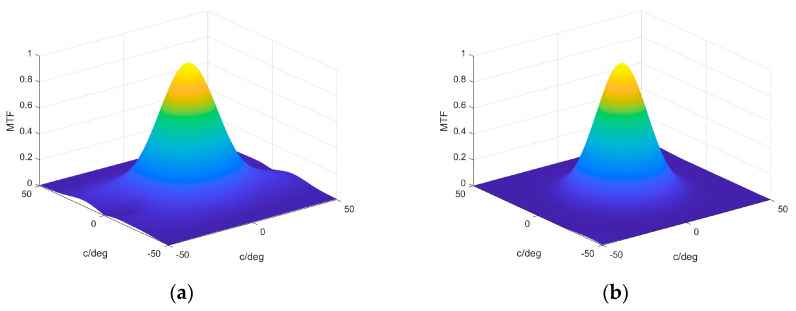
The MTF obtained from the wave aberration function for (**a**) transmission and **(b**) reflection recording geometries in Figure 1.

Following this analysis and comparison, the corresponding MTF is presented in Figure 11. In this case, we can estimate the cutoff frequency and compare it with the experimental values obtained and their comparison with the minimum value given by the diffraction limit. The value obtained in Figure 11a for each axis is around 40 cycles/degree, which is half that obtained for asymmetrical lenses recorded in [34] with the Biophotopol photopolymer and double that obtained for symmetrical lenses with the same recording material. The MTF form in Figure 11b presented a more symmetrical form and a slightly smaller cutoff frequency, around 35 cycles/degree in both main axes.

**Figure 12:**
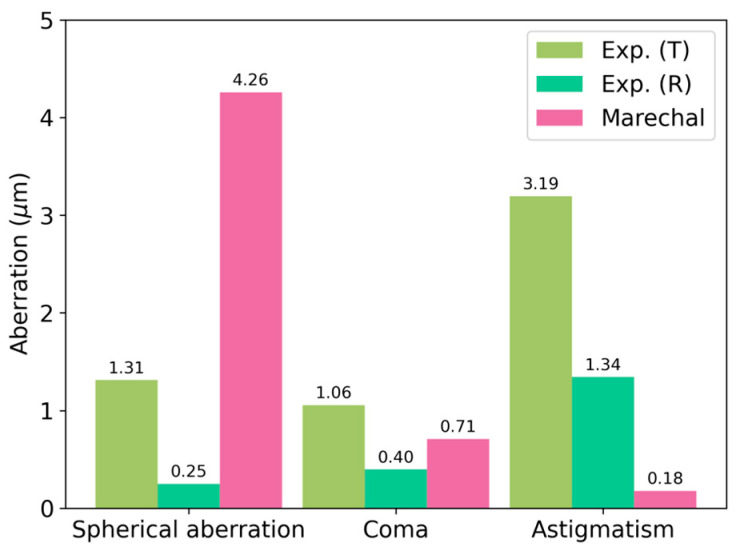
A comparison of the Seidel coefficients with the Marechal tolerances for transmission and reflection recording geometries in Figure 1.

From the wave aberration function, we can compute the Seidel coefficients for the aberration terms that are relevant for our purpose (spherical aberration, coma, and astigmatism) and compare them with the so-called Marechal tolerances [34], which depend on the wavelength of the reconstructed beam. The experimental results and their comparison are shown in Figure 12.

We point out that the astigmatism contribution is considerable in both cases and that every term is reduced when measuring the reflection HL.

## 4. Conclusions

In this work, we described the fabrication of HLs that are useful as wave couplers recorded without prisms using transmission and reflection geometries. We studied the limitations of this method of fabrication, and then we proposed a simple model to estimate the new focal length when the wavelength is shifted and therefore consider the shrinkage effects in the photopolymer film during the recording or curing process. Furthermore, we recorded these HLs with high values of diffraction efficiencies in both geometries. Finally, we analyzed the quality of these lenses and obtained good results, equivalent to the HLs designed for other applications, where the light is guided along the substrate as the focalization scheme dictates.

## Figures and Tables

**Table 1:** Specific values for the transmission HL for a given diameter of 20 mm and original focal length in air of 80 mm: central grating parameters, diffracted beam angles inside the material, and focal length as a function of shrinkage and the readout wavelength $\lambda_{c}=\;633\;nm$

Shrinkage (%)	Central Grating		Diffracted Angles Inside (°), $\lambda_{c}$ = 633 nm			Focal Length Along the Substrate (mm)
	Slanted Angle (°)	Spatial Freq. (lines/mm)	Highest	Central	Lowest	
0	−68.7	1758	43.9	43.0	41.9	370.9
1	−68.5	1760	44.1	43.2	42.1	369.2
2	−68.3	1763	44.4	43.4	42.3	367.5
4	−68.0	1768	44.9	43.9	42.8	364.1
8	−67.1	1777	45.7	44.8	43.6	357.3

**Table 2:** Specific values for the reflection HL for a given diameter of 20 mm and original focal length in air of 80 mm: central grating parameters, diffracted beam angles inside the material, and focal length as a function of shrinkage and the readout wavelength $\lambda_{c}=\;473\;nm$.

Shrinkage (%)	Central Grating		Diffracted Angles Inside (°), $\lambda_{c}$ = 473 nm			Focal Length Along the Substrate (mm)
	Slanted Angle (°)	Spatial Freq. (lines/mm)	Highest	Central	Lowest	
0	−27.9	5596	−57.1	−56.3	−55.4	301.5
1	−27.7	5640	−56.1	−55.3	−54.4	301.2
2	−27.4	5684	−55.0	−54.2	−53.2	300.2
4	−27.0	5772	−52.7	−51.9	−50.9	295.6
8	−26.1	5949	−47.9	−46.9	−45.9	274.8

## Data Availability

The original contributions presented in this study are included in the article. Further inquiries can be directed to the corresponding author.

## Abbreviations

The following abbreviations are used in this manuscript:

TIR	Total Internal Reflection (Principle)
HL	Holographic Lens
HS	Hartmann–Shack (Sensor)
CW	Coupled Wave (Theory)
MTF	Modulation Transfer Function
